# What do families value most about the care home where their older adult relatives live?

**DOI:** 10.3389/fpubh.2024.1338649

**Published:** 2024-08-08

**Authors:** Eduardo Alberto Leché-Martín, María Isabel Saz-Gil, Ana Isabel Gil-Lacruz, María José Sierra-Berdejo, Marta Gil-Lacruz

**Affiliations:** ^1^Servicios Sociales Comunitarios, Ayuntamiento de Zaragoza, Zaragoza, Spain; ^2^Departamento de Dirección y Organización de Empresas, Facultad de Ciencias Sociales y Humanas, Universidad de Zaragoza, Teruel, Spain; ^3^Departamento de Dirección y Organización de Empresas, Escuela de Ingeniería y Arquitectura, Universidad de Zaragoza, Zaragoza, Spain; ^4^Departamento de Psicología y Sociología, Facultad de Ciencias Sociales y del Trabajo, Universidad de Zaragoza, Zaragoza, Spain; ^5^Departamento de Psicología y Sociología, Facultad de Ciencias de la Salud, Universidad de Zaragoza, Zaragoza, Spain

**Keywords:** care homes, evaluation of services, psychosocial indicators, family involvement, corporate social responsibility, healthy organizations

## Abstract

**Introduction:**

The 2030 Agenda and the principles of Corporate Social Responsibility (CSR) define companies and public authorities as agents for social change sharing objectives such as promotion of health, personal development and social engagement, among others. Care homes for the older adult are an example of organizations that should be particularly aware of these priorities. Since they work with vulnerable groups, collaboration with the families is essential in ensuring residents' wellbeing.

**Methods:**

The objective of this study is to analyse the factors that condition the satisfaction of relatives of residents in a care home for the older adult located in a rural environment in the province of Huesca (Spain).

**Results and discussion:**

The 51 relatives interviewed rated the following points very positively: location and accessibility, food service, medical resources, communication with the staff and management team. A high percentage, however, did not know about the channels for volunteer work and institutional involvement. Some psychosocial indicators related to families' interaction and communication with the staff and their potential involvement in the dynamics of the institution have considerable weight in how they explain their satisfaction. These results may lead to new lines of research and intervention that contribute to improving the quality of this type of resources and their commitment to the Sustainable Development Goals (SDGs) and social responsibility.

## 1 Introduction

The implementation of the 2030 Agenda and the Sustainable Development Goals (SDGs) represent a solid commitment to social inclusion (1), and both are closely linked to the integration of the older adult and people with disabilities. This is evidenced by the goals that promote health and wellbeing for all ages (SDG 3), creating inclusive, sustainable, safe and resilient societies (SDG 11), and promoting peaceful and inclusive societies (SDS 16).

The approach to accomplish these objectives is based on the commitment to a methodology of consensus and collaboration between the government, the public authorities, the private sector and society as a whole (2). In this vein, the UN Global Compact represents an international initiative that promotes sustainable development and corporate social responsibility (CSR). The pact was signed in 2000 by more than 13,000 organizations in over 170 countries and since 2015, it has been committed to promoting the SDGs in the business sector, raising awareness among companies and organizations and helping them, as these are a key factor for social change (1).

Being a socially responsible company implies the implementation of strategic planning encompassing the economic, social and environmental aspects of business activity (3). In 2011, the European Commission added the requirement that companies should integrate ethics, respect for human rights and consumer concerns (4). The adoption by companies of the principles of CSR offers several advantages; for instance, it promotes sustainable development, improves corporate public image, increases stakeholder satisfaction and loyalty and, consequently, increases the organization's value (5).

This concept, however, is controversial, as is the case with the SDGs, since it runs the risk of becoming a new trend lacking in substance due to excessive and widespread use. These shortcomings may be particularly obvious when working with the older adult, who are often not considered, for example, in implementing the SDGs (6). Several measures could redress this unresolved issue: providing specific training for professionals in caring for the older adult, ensuring sustainable architecture in care facilities, adapting the equipment, integrating their services into the community, etc. Conducting research into the analysis of the individual and organizational factors underlying the active involvement of older adult people is also a challenge that should be addressed by programmes on healthy aging (7).

There is need for a firm commitment to involving the older adult and the people close to them, and this should begin by improving their immediate surroundings. In this sense, care homes may represent a unique opportunity to implement such democratizing dynamics (8). Given that care homes are, and will continue to be, one of the most frequent options for senior living, finding a way to guarantee their quality is a systemic objective of local and macro-level public policies.

In Aragón (Spanish Autonomous Region), for instance, Title VI of Law 5/2009, of 30 June, on Social Services in Aragón, article 60 (9), states that: “In all public centers that provide social services or where social activities are conducted and in private centers that are publicly funded, there should be procedures regarding the participation of the users or their legal representatives in the running of the center or service, as determined by the regulations”. Relatives, as representatives of users of the Public System of Social Services, may participate in the planning and development of programmes and activities, according to article 9 of this law, exercising their right, among others, to be involved in the center's decision-making processes and to provide appropriate evaluations on its functioning. Furthermore, according to article 15.2, they may inform the relevant authorities of any infringement of these rights, through the established body for participation (9).

As quality management systems, regional legislation is complemented by the implementation of the standards set by the International Organization for Standardization (IOS), specifically, the Una Norma Española (UNE—One Spanish Standard) 158001 standard on Services for the Promotion of Personal Autonomy, Management of Residential Care Homes (Asociación Española de Normalización, AENOR 2007—Spanish Association for Standardization and Certification) (10), which includes the minimum requirements that residential centers need to meet in order to ensure quality of service.

These requirements, evaluated by indicators, explicitly address families' collaboration, regarding the intervention itself and the internal evaluation processes—by operationally measuring their participation in scheduled activities, their overall satisfaction or their specific complaints about the establishment.

These models, however, do not measure all the aspects of quality care, as they are mainly geared to medical and health-related issues and to the organization and management of human resources (11). Even though IOS standards consider indicators relating to processes, internal communication, employee motivation, etc. (12), they background the evaluation of other issues such as interpersonal relationships between the different members in the system. Therefore, psychosocial indicators that influence families' satisfaction with the health and lay professionals at the care homes are left out.

This need for improvement in residential care, for institutional transparency and for consideration of family involvement poses new organizational challenges. Along these lines, the *Asociación Estatal de Directores y Gerentes en Servicios Sociales* (Spanish State Association of Social Services Directors and Managers) (2021, p. 9) (13) outlines a new model of residential care in which: “families are key actors, with no limits to their visits, hours and areas”. The emphasis is on encouraging the continuity of family life to strengthen residents' emotional wellbeing by contributing “resolutely to the openness and transparency of the centers and their homelike atmosphere so that residents do not feel institutionalized, but rather view the center as their home, a place where they can continue their family life and life plan”.

This new paradigm of care focusing on the person and their individual preferences aims to improve residents' wellbeing and relatives' satisfaction (14, 15). Therefore, encouraging collaboration and promotion of meaningful bonds—with the family, close friends, neighbors, etc.—is also in line with the SDGs and the promotion of healthy and successful aging (16).

Although families' satisfaction with and opinion of residents' quality of life, attention and care are considered important, few studies in the literature have analyzed the topic in depth. The review of the state of the art reveals that these aspects have been backgrounded both as long-term criteria for evaluation of care homes and in terms of their value as basic indicators of the CSR of said institutions (17–20).

An involved family can supply relevant information about the quality of the care provided to their relatives from valuable and unique perspectives; this offers the added advantage of providing a person-centered measurement of quality (21, 22). Factoring in relatives' opinions contributes significantly to improving the evaluation, design and implementation of strategies and resources at care homes (23). For this reason, and in view of the progressive aging of the population, research studies that collect first-hand opinions from relatives about residents' care and create specific baselines for research and intervention are becoming increasingly important (17, 24).

This line of research should be used with caution. The information provided by families may have limitations, as some responses may be conditioned by previous personal experiences or conflicting emotions or may even involve social desirability bias; therefore, a system based on sufficient, reliable indicators to offset the errors and contradictions that may arise would be valuable (25). This premise involves transcending the use of single-dimension indicators—such as overall satisfaction with the center—and aiming for the inclusion of other indicators within a multidimensional model (26).

In their recent systematic review, Rodríguez-Martínez et al. (27) observe that certain indicators that evaluate nursing home facility characteristics can be utilized by both residents and their kin, thereby simplifying their comparison and corroborating their utility as instruments for quantifying the quality of life of the residents.

One way to classify common indicators is to distinguish between the assessment of the nursing home's physical characteristics (such as its location, facilities, and bedrooms) and the characteristics of its staff (including their training and the quantity and quality of care they provide) (28). Another model, developed by Shippee et al., identifies the following common domains: care attention, staff involvement, environment conditions, and food enjoyment (21).

Rodríguez-Martínez et al. (p. 10) (27) integrate a more comprehensive assessment system that encompasses three key categories: (a) structural features, (b) financial resources or payments, and (c) staffing and administrative resources. The proposed system is based on the contributions of several authors (29–31).

Structural characteristics comprise of various indicators such as ownership of the residence (public-private), location (rural-urban), and size of the residence quantified by the number of beds, chain affiliation, and the health status of residents (29, 32). Within the category of structural characteristics, three types of indicators should be taken into consideration: space management, building services, and supporting facilities (27).

The assessment of environmental features related to access (i.e., visual differentiation, signage and layout in the care home) may be particularly important for vulnerable groups with impaired visual and orientation skills. In particular, visual differentiation is positively associated with orientation, regardless of the group (residents, family carers and staff), while signage and layout contribute to a better orientation experience, especially for older residents (33).

Staffing and administrative resources refer to the quantity and quality of human resources, their commitment to the residents, level of expertise, and staff turnover (34). In relation to the indicators used in the family assessment of healthcare workers, Cook et al. used a combination of a questionnaire (Nurse Practitioner Satisfaction Survey) consisting of three subscales (satisfaction, communication, and accessibility) and a focus interview whose themes were: Care Coordination, Prevention of Acute Care Utilization, and Access to Care (35).

These studies are relatively new and have an Anglo-Saxon origin. Further research is needed to establish the generalisability of their findings and determine the significance of each indicator on the satisfaction of nursing home users.

Our study proposal involves examining these categories in a multidimensional model with a special emphasis on psychosocial indicators that promote family involvement and functioning. We aim to measure family satisfaction in areas such as facilities, services and activities, care and support, staff communication and leadership, as well as channels for formal and informal participation.

## 2 Materials and methods

The operational study of the assessment and the degree of satisfaction of relatives of residents in a care home was based on the analysis of center located in a municipality with a registered population of 9,352 inhabitants in 2021 (province of Huesca, Spain).

In 2018, this state-funded, privately run care home had 92 beds and 27 day-center places. The care home's guiding principles include individualized care for residents, the prevention of polypharmacy, the home's involvement in the community (e.g., a specific intergenerational programme), and the development of its staff's continuing training and professional wellbeing. It has activity programmes: meaningful, recreational and cultural activities, cognitive stimulation, memory, functional rehabilitation, and physical exercise. It also has a cafeteria, a chapel, private and specialized areas, a multi-purpose room, a garden and a car park. The most common room type is double occupancy.

Approximately two thirds of the residents are over 80 years old and their cognitive abilities are not impaired, although there is some functional diversity in terms of mobility, visual impairment and presbyacusis. The predominant profile is of women living alone, either because they are widowed or because they remain single. Most of the residents were born and lived in the region, so they have an important link with the town where the care home is located.

Based on information from the administrative staff, the majority of families live in the vicinity of the residence and maintain frequent communication and visitation with their loved ones residing in the residence. The family members consist mainly of sons and daughters of the residents, followed by nieces, nephews, and siblings. On average, the age of relatives is over 50 years old.

This study involved voluntary and anonymous participation by 51 relatives of residents. The only requirement for participating in the interview was being a relative of a resident in the center—that is, a non-probability convenience sampling procedure. Prior to the interview, the subjects were explained the objectives and their informed consents were requested.

Once the sample had been defined, the focus shifted to data collection, which consisted of applying a questionnaire in the care home during morning and afternoon visiting hours. The interview was conducted in person by a properly trained social worker from the research team. The interview spanned 1 month and lasted approximately 25 min. The activity was approved by the care home, which facilitated the circulation of information about the study and provided a space to conduct the interviews in privacy.

In order to ensure the reliability of the questionnaire, it was based on the instrument created by a research group that focus its activity on the social services for the Alto Gállego region, due to the suitability of the indicators and the geographical proximity of its area of activity. The initial version of this questionnaire comprises sections devoted to resident care, company management, and the satisfaction levels of relatives regarding the services, attention, and care that users receive.

After making the appropriate revisions and modifications, the final questionnaire was subjected to an expert peer review by the multidisciplinary research group Wellbeing and Social Capital, with ref. S16_23R, from the University of Zaragoza and the Regional Government of Aragón.

The instrument poses questions with two types of response: dichotomous and ordinal (on a Likert scale). Most of questions related to relatives' satisfaction with the care come conditions and services, have five response options: very unsatisfactory (coded 1), unsatisfactory (coded 2), indifferent (coded 3), satisfactory (coded 4) and very satisfactory (coded 5).

The different indicators used are grouped as follows: (a) environmental dimensions, such as location, access and surroundings; (b) the care received, in terms of activities, services or equipment available; (c) communication within and outside the care home; and (d) family involvement.

The SPSS-20 statistical package has been used to build the database and perform the statistical analysis. After exploring the descriptive results, Pearson's correlation was used for statistical analysis, as it examines the relationship between two numerical variables. Finally, in order to synthesize the results, two analyses were conducted: the “overall evaluation of the care home” variable was analyzed using Kruskal-Wallis test and the “recommendation of the care home to other people” variable using Chi-square test.

## 3 Results

### 3.1 Descriptive results

Descriptive results are shown in Table 1.

**Table 1:** Sociodemographic characteristics and satisfaction level with the main care home characteristics answered by the relatives' sample.

**Variable**	**Characteristics**	**Frequency (*N* = 51)**	**Percent**
Age	20–49	6	11.8
	50–69	37	72.6
	<70	8	15.6
Sex	Male	16	31.4
	Female	35	68.6
Living in the residence localization	Yes	44	86.3
	No	7	13.7
Family relationship with the resident	Couple	2	3.9
	Daughter/son	32	62.7
	Sister/brother	3	5.9
	Nephew/niece	8	15.7
	Other	6	11.8
Care home visit frequency	Every day	29	56.9
	Several days per week	2	3.9
	Once per week	14	27.4
	Several days per month or in a special occasion	6	11.8
Contact with the resident without visit him/her	Every day	1	2
	Several days per week	3	5.9
	Several days per month or in a special occasion	26	51
	Never	20	39.2
Satisfaction with the care home's location	No/indifferent	4	7.8
	Yes	47	91.4
Satisfaction with the care home's access	No/indifferent	2	4
	Yes	49	96.1
Satisfaction with the mobility aids for people with reduced mobility	No/indifferent	6	11.7
	Yes	45	88.2
Satisfaction with common areas	No/indifferent	13	25.5
	Yes	38	74.5
Satisfaction with the cleanliness status of the residence	No/indifferent	24	47
	Yes	26	51
Satisfaction with room facilities	No/indifferent	16	31.4
	Yes	33	64.7
Satisfaction with the range of services at the care home	No/indifferent	11	23.6
	Yes	26	51
Satisfaction with the quality and variety of services at the care home	No/indifferent	14	27.4
	Yes	23	45.1
Satisfaction with food service	No/indifferent	17	33.4
	Yes	33	64.7
Satisfaction with the medical and nursery services	No/indifferent	12	23.5
	Yes	36	70.6
Satisfaction with the laundry service	No/indifferent	26	51
	Yes	21	41.1
Satisfaction with the resident attention	No/indifferent	15	29.5
	Yes	36	70.6
Satisfaction with the communication between professional staff and relatives	No/indifferent	14	27.4
	Yes	32	62.8
Satisfaction with the speed of the staff in finding solutions	No/indifferent	14	29.4
	Yes	29	52.9
Satisfaction with the swift processing complaints and suggestions	No/indifferent	13	25.5
	Yes	21	41.2
Satisfaction with the management team and their work	No/indifferent	16	31.3
	Yes	32	62.7
Knowledge about participation in the care home	No/indifferent	49	96.1
	Yes	2	4
Satisfaction with options to be volunteer	No/indifferent	46	90.2
	Yes	5	9.8
Satisfaction with the care home volunteers	No/indifferent	6	11.7
	Yes	33	64.7
Satisfaction with the community integration	No/indifferent	13	25.5
	Yes	32	62.8
Satisfaction with price-quality ratio	No/indifferent	18	35.3
	Yes	28	54.9
Satisfaction with the amount of staff	No/indifferent	46	90.2
	Yes	4	7.9
Overall evaluation of the care home	From 0 minimum to 4	3	5.9
	From 5 to 6	18	35.3
	From 7 to 10	29	56.8
Care home recommendation to other persons	No/indifferent	15	29.5
	Yes	36	70.5

#### 3.1.1 Sociodemographic characteristics

Of the 51 relatives participating in the study, over half are women (69%), aged between 50 and 69 years old (72%) and living in the residence localization (86%). The most frequent family relationship is that of daughter/son of residents (63%). These data reveal the close connection with residents.

#### 3.1.2 Visits to residents

Of all the families participating in the study, 57% make daily visits to residents and 27% do so at least once a week. Therefore, according to the relatives, there is little need for contact by telephone or other means of communication.

The care home's location and accessibility contributes to the high frequency of visits. Of all the respondents, 91% are “satisfied” or “very satisfied” with the location, 96% feel similarly about its accessibility, including the road that leads to the care home and the car park, the access infrastructures, and mobility aids for people with reduced mobility (88% of respondents).

Families have a “very satisfactory” or “satisfactory” perception (70%) of the condition of and access to the common areas. Of all the respondents, 59% think that the characteristics of the bedrooms (dimensions, lighting, and furniture, among others) contribute to a pleasant stay for residents.

#### 3.1.3 Satisfaction with the services offered by the care home

Of all the families that make up the sample, 51% describe the range of services at the care home as “satisfactory” or “very satisfactory”, compared to 9% that think the opposite (“unsatisfactory” or “very unsatisfactory”).

These data are similar in the evaluation of the quality and variety of the tasks included in the services, as 45% of respondents rate them as “satisfactory”, as opposed to 11% who express their dissatisfaction.

The results reveal that the food and medical services are the most highly rated aspects, with 65% of the sample in both cases perceiving them as “very satisfactory” or “satisfactory”.

Families rate positively the food service in 65% of cases, in contrast to the 6% who rate it negatively. The significant percentage of families who are indifferent to this service is noteworthy (28%).

Regarding the medical service, 65% of family members give a positive evaluation for the care received by their relatives, as well as the monitoring and medical reports they are provided with.

With respect to the laundry service, 1 in 3 respondents express indifference and 17% find it “rather unsatisfactory”.

Half of the relatives consider that the care home has enough courses and workshops to make residents' leisure time pleasant.

#### 3.1.4 Relationship between the care home staff and relatives

Of all the relatives in the sample, 41% find that the attention they receive from the care home staff and the treatment of residents is “very satisfactory”, with fewer than 10% of the ratings being negative.

Along these lines, the study reveals high levels of satisfaction with the communication between professional staff and relatives, with 63% of cases perceiving this as “very satisfactory” or “satisfactory”.

The speed of the staff in finding solutions and adopting measures in the event of conflicts, and in informing the families promptly is also of note, with 30% of relatives rating these as “very satisfactory” and 27% as “satisfactory”.

Similarly, families are aware of the swift processing of their complaints and suggestions by the care home managers. The management team and their work are rated as “satisfactory” or “very satisfactory” by 63% of the respondents.

#### 3.1.5 Involvement in care home life

Regarding involvement in decision-making bodies or activities, the study reveals that 96% of relatives report lack of knowledge about both this possibility and the regulatory procedures that guide it.

The findings are similar for the processes of involving relatives as volunteers (90% of respondents did not know that they could do it). Of all the families in the sample, 65% view as “satisfactory” having external volunteers at the care home.

Finally, 63% of respondents perceive as positive the integration and involvement of residents in their surroundings.

#### 3.1.6 Overall evaluation of the care home

The aspects most highly valued by relatives are: location of the care home, price-quality ratio and professionalism of the employees. These aspects lead to an average overall evaluation of 7 points on a 10-point-scale. In the same vein, 7 out of 10 respondents would recommend the care home.

Despite the level of satisfaction reflected, the respondents declare that the number of professionals in the care home should increase in order to offer better service and more personalized care.

### 3.2 Inferential results

After analyzing the descriptive results, this study delves into the statistical relationship between the variables.

Firstly, the analysis focuses on the number of correlations of all the variables grouped by sociodemographic dimensions with the indicators of satisfaction with the care home (location and access, equipment, services and activities, care and support, communication, participation and overall evaluation) (see Table 2). Secondly, the two variables with a large number of correlations and also a close relationship in terms of behavior prediction (evaluation and recommendation of the care home) are selected for independent, detailed analysis. Lastly, Kruskal-Wallis test is run with the first of these variables to obtain a synthesis equation for the main results. Subsequently, a Chi-square test is conducted with the recommendation of the care home variable. Two different inferential statistical procedures are used to confirm the validity of the results.

**Table 2:** Correlation between families' evaluation and recommendation of the care home with the care home characteristics.

**Care home characteristics**		**Families evaluation of the care home**	**Recommendation of the care home**
Frequency of visits	r	−0.31^**^	−0.42^**^
	*p*-value	0.029	0.008
	N	50	39
Location of the care home	r	0.39^**^	
	*p*-value	0.005	
	N	50	
Access for people with reduced mobility	r	0.36^**^	
	*p*-value	0.011	
	N	50	
Common areas	r	0.46^***^	
	*p*-value	<0.001	
	N	50	
Care home cleanliness	r	0.61^***^	
	*p*-value	<0.001	
	N	49	
Furniture	r	0.39^**^	
	*p*-value	0.005	
	N	50	
Sense of safety	r	0.45^***^	
	*p*-value	<0.001	
	N	47	
Bedroom facilities	r	0.57^***^	0.33^**^
	*p*-value	<0.001	0.045
	N	48	37
Food service	r	0.58^***^	0.35^**^
	*p*-value	<0.001	0.033
	N	49	38
Laundry	r	0.66^***^	0.54^***^
	*p*-value	<0.001	0.001
	N	46	36
Medical service and nursery	r	0.66^***^	0.49^***^
	*p*-value	<0.001	0.002
	N	48	37
Medical information provided to families	r	0.63^***^	0.45^**^
	*p*-value	<0.001	0.006
	N	44	47
Individual care and support	r	0.58^***^	
	*p*-value	<0.001	
	N	50	
Monitoring of the residents	r	0.69^***^	
	*p*-value	<0.001	
	N	47	
Number of professionals	r	0.46^**^	
	*p*-value	0.001	
	N	49	
Residents attention	r	0.58^***^	
	*p*-value	<0.001	
	N	50	
Family attention	r	0.61^***^	
	*p*-value	<0.001	
	N	50	
Reference staff whom to talk	r	0.57^***^	0.36^**^
	*p*-value	<0.001	0.023
	N	50	39
Staff accessibility	r	0.59^***^	
	*p*-value	<0.001	
	N	50	
Management collaboration	r	0.68^***^	0.35^**^
	*p*-value	<0.001	0.034
	N	47	37
Communication among staff and resident	r	0.71^***^	0.45^**^
	*p*-value	<0.001	0.008
	N	41	34
Communication among staff and family	r	0.66^***^	0.35^**^
	*p*-value	<0.001	0.035
	N	45	36
Resolution of problems	r	0.70^***^	0.43^**^
	*p*-value	<0.001	0.013
	N	44	33
Complaints resolution	r	0.72^***^	
	*p*-value	<0.001	
	N	34	
Activities and workshops offered	r	0.47^**^	
	*p*-value	0.003	
	N	38	
Services 'reliability and diversity	r	0.50^**^	
	*p*-value	0.002	
	N	37	
Services quality	r	0.41^**^	
	*p*-value	0.008	
	N	42	
Price-quality ratio	r	0.56^***^	0.378^**^
	*p*-value	<0.001	0.027
	N	45	34
Residents options of taking decisions about care home	r	−1^***^	
	*p*-value	<0.001	
	N	2	
Family options of care home participation	r	−0.606^**^	
	*p*-value	0.037	
	N	12	
Family users committee	r	−1^***^	
	*p*-value	<0.001	
	N	2	
Integration in the town	r	0.67^***^	0.43^**^
	*p*-value	<0.001	0.01
	N	44	35
Overall care home evaluation	r	1	0.64^***^
	*p*-value		<0.001
	N	50	39

^**^Statistically significant at p < 0.05, ^***^Statistically significant a p < 0.001.

The number of positive correlations between the communication dimension and the other variables is of particular interest, specifically, access to staff, collaboration, communication between professionals, residents and families, and processing of problems and complaints and swiftness in finding solutions. Likewise, there is an evident lack of correlations with the complaints box and volunteering variables.

The correlations obtained with the overall evaluation of the care home and the likelihood of recommending it to other people are analyzed individually given their descriptive weight.

#### 3.2.1 Correlations with the families' evaluation of the care home

The overall evaluation of the care home is the variable with the larger number of statistically significant correlations and a greater variety of dimensions.

The location of the care home, access for people with reduced mobility or integration in the town are reasons for a positive evaluation of the care home by relatives. In contrast, families' evaluation of the frequency of their visits is negative.

Elements of the care home such as common areas, furniture, bedroom, cleanliness of these elements, or sense of safety, are reasons for the overall satisfaction of the relatives with the center.

In this positive trend, services such as food service, laundry, workshops or activities, medical service, medical information provided to the families, perception of these services' reliability and quality are noteworthy.

In contrast, in terms of life at the care home, opinions are less favorable concerning the possibility of being involved, both for residents and families, and the functioning of the users' committee.

However, the overall evaluation of the care home has a positive correlation with the positive evaluation of the care provided by the lay staff. In this regard, correlations are positive regarding individual care and support, satisfaction with the treatment of residents and families, accessibility and monitoring of users.

Regarding human resources, the relatives exhibit overall satisfaction with the care home in terms of the number of professionals, communication with residents and families, knowing whom to talk to at all times, or the sense of a real and direct possibility of talking to the management.

Furthermore, the ease with which complaints are processed, the swift and flexible resolution of problems or the price-quality ratio of the care home are also the basis of a positive evaluation of the center overall.

#### 3.2.2 Correlations with recommendation of the care home

The favorable evaluation of the care home is associated with relatives' recommendation of the center to other people. This recommendation presents an inverse correlation with the frequency with which relatives can visit.

Other relevant aspects connected to recommending the care home are its integration in its immediate surroundings, the spaces and services provided such as the bedroom, the food service, the laundry, medical and nursing human resources and the information relatives receive about the patient's health.

The positive evaluation of the team of professionals is another of the positive associations when suggesting this care home to other people. Staff communication with residents and families, knowing whom to talk to or the information received from the management team are of particular relevance. Additionally, the swift resolution of problems or the adequate price-quality ratio are also conducive to the recommendation of this care home service.

#### 3.2.3 Kuskal-Wallis test about the evaluation of the care home

Kruskal-Wallis test is a non-parametric tool used to determine whether there are statistically significant differences between the medians of three or more independent groups. In this research, we selected as dependent variable: the overall assessment of the residence and as independent variables the main characteristics of the residence that have obtained positive results in the previous correlation test.

The original variables relating to the evaluation of the care home that have been introduced into the model are: access for people with reduced mobility, access to staff, comfort of the bedroom, residential care and support, general quality, price-quality ratio, variety of services, family communication, communication with staff, communication with the management team, access to care home information, cleanliness of the care home, volunteer participation, complaints, knowing whom to talk to in case of need, user care and support, common areas, location of the care home, solution of problems, security, food service, laundry service, medical service and variety of services (see Table 3).

**Table 3:** Kruskal-Wallis Test among evaluation of the care home and care home facilities.

**Care home facilities**	***N***	**Kruskal-Wallis**	**gl**	***p-*valor**
Access for people with reduced mobility	51	11,886	7	0.104
Access to staff	51	23,209	7	0.002
Bedroom facilities	49	18,161	7	0.011
General quality	42	16,804	6	0.010
Price quality ratio	46	21,023	7	0.004
Variety of services	37	12,021	7	0.100
Family communication	46	21,775	7	0.003
Health care staff communication	44	22,386	7	0.002
Management staff communication	48	23,102	7	0.002
Access to care home information	47	24,831	7	0.001
Cleanliness of the care home	50	20,043	7	0.005
Volunteer participation	39	5,656	7	0.580
Complaints solution	34	21,812	7	0.003
Knowing whom to talk in case of need	51	20,372	7	0.005
User care and support	51	18,322	7	0.011
Common areas	51	14,080	7	0.050
Location of the care home	51	15,959	7	0.025
Solution of problems	44	23,486	7	0.001
Security	48	18,210	7	0.011
Food service	50	20,576	7	0.004
Laundry service	47	20,931	7	0.004
Medical service	48	22,552	7	0.002
Number and variety of services	38	10,471	7	0.163

#### 3.2.4 Care home recommendation Chi-square test

The Chi-square test has been selected because it is considered appropriate to determine whether there is a significant association between a nominal dependent variable (recommendation or not recommendation the care home) and several ordinal independent variables, such as satisfaction with different aspects of a care home.

Table 4 shows the main results obtained: *p*-value, the number of people interviewed who would recommend the care home and their evaluations about the care home conditions and services. Results indicate that the independent variables have a relevant impact on the indicator “recommendation of the care home” and, therefore, statistically significant differences are inferred.

**Table 4:** Chi-square test conducted between the recommendation of the residence and the rating of the various characteristics of the residence.

**Name of variable/ characteristics of the care home evaluated**	***N***	**Relatives who would recommend the residence being satisfied with the characteristics of the care home, Number (%)**	**Chi-squared *p*-value**
Features of the bedroom	37	26 (76.5)	0.001
Food service	38	28 (80)	0.004
Laundry service	36	20 (60.6)	0.002
Medical and nursing services	37	29 (82.8)	<0.001
Resident information and monitoring	36	26 (76.5)	<0.001
Knowing whom to talk to	39	35 (97.2)	0.006
Management information and collaboration with families	37	30 (83.3)	0.02
Communication between professionals and residents	34	29 (84.8)	0.001
Communication with relatives	36	27 (77.2)	0.042
Solutions to problems found as quickly as possible	33	26 (81.3)	0.001
Price-quality ratio	34	23 (69.7)	0.001
Integration of the care home in the town life	35	29 (87.9)	0.006
Care home evaluation	39	34 (94.4)	<0.001

Families' evaluation of the care home has great statistical significance regarding the recommendation of the care home. The explanatory weight of the level of satisfaction with the laundry service and the medical service when recommending these resources is also of note.

The variables knowing whom to talk to, price-quality ratio, communication with the family, and those associated with specific aspects such as food service and the features of the bedroom have a direct influence on whether to recommend the care home or not, although they have a lower statistical significance than the above-mentioned points.

## 4 Discussion

As reported by other studies (21, 22, 24), the results of this research indicate the need to consider the opinion and level of satisfaction of relatives, as both are closely related to residents' wellbeing. Furthermore, satisfaction and family involvement are themselves positive indicators in the evaluation of residential care homes (23, 26).

In our case, the perception of relatives' satisfaction with the care home depends on several dimensions, such as location, price-quality ratio and the professionalism and attention shown by the employees. Families' satisfaction is also positively linked to the services provided, such as the food, medical and laundry services.

The scientific literature shows a seemingly contradictory trend. In Canadian studies, residents reported higher quality of life in rural areas (36). However, this rural assessment is not consistent and is worse for their relatives in other contexts (21). In Minnesota, relatives preferred the living conditions and characteristics of suburban care homes, especially in relation to care, environment and food, but less so in relation to staff (21).

It would be interesting to analyse how these ratings might be influenced by other factors such as proximity and ease of access to facilities, price, perceptions of professional expertise, etc. In our case, the fact that the residents and their relatives born or lived in the region, this home care is a public-private resource and the residence is well communicate, influence positively in the family assessments. This finding may also be consistent with the importance of maintaining meaningful relationships and community links for older residents (in this case in their own home village) (37).

Among the different items analyzed, two have been selected for their importance and high number of correlations with the different dimensions evaluated: the relatives' evaluation and recommendation of the care home.

The variables with the most influence on families' evaluation of the care home are: (a) collaboration and communication between the medical service and the family, (b) support and information given to family members by the management, and (c) access to the care home for people with reduced mobility.

Special mention should be made of the importance given to the issue of access to the care home. This is an issue that for the resident can encompass a number of important indicators e.g., accessibility, nonslip floor, adaptations to doors and windows, handrails, signage and so on (31). Family members agree on the importance of this dimension in the quality of life and functional autonomy of residents (33).

The importance of the staff-related characteristics dimension in family satisfaction would be congruent with the results obtained in scientific literature. In the study by Shippee et al., it is also observed that even the family assessment of a single item in the professional dimension, staff retention, has an impact on the assessment of other variables such as care, the staff as a whole and the care home environment (21).

These results show a similar pattern to the evaluations expressed by the residents themselves and older adult people in general: the aspects relating to the social context—atmosphere, interaction, treatment, etc.—play a major part in the evaluations of the resources and services they are offered (38).

The Chi-square test conducted on the recommendation of the care home shows that this perception is directly linked to the positive co-operative relationships between the relatives and the professionals in charge of healthcare and management and to the overall evaluation of the center, the price-quality ratio and specific resources such as laundry, food service, etc. The relatives stated that they would recommend the care home based on the guarantee of its basic and essential services—which are also intimate and personal—and the features of the bedroom or the food service.

The common trend of these results points to the psychosocial aspects of collaboration with the family by the center's management and medical services. Therefore, communication is a fundamental indicator of perceived quality and includes several aspects relating to the care and support of residents and their relatives (e.g., monitoring residents, communication between residents and professionals, access to the staff, swiftness of finding solutions to problems and complaints, etc.). In addition, the restrictions and stress generated by the pandemic among residents and their families has placed the importance of maintaining continuity, transparency and collaboration between families, staff and management as a priority (39).

This means that the evaluation of the care home involves both objective and subjective factors. Care home quality models that factor in relatives' opinions should consider that their satisfaction is a multidimensional construct encompassing key issues such as the center's accessibility, provision of services and resources, personalized care and the communication established with professionals (including the management and medical teams). These factors suggest the convenience of implementing the model of personal care and residential center/home (14, 15, 23). Therefore, the center becomes a homelike place where the members of the family are key actors. In short, the aim is to ensure a new paradigm of comprehensive care and support geared to the people and their personal needs and preferences while also valuing the importance of the family's role.

The results of this study agree with the scientific literature in highlighting that the evaluation of the level of user satisfaction is an adequate strategy to estimate the quality of an intervention programme (40). Quality systems, such the UNE standard for the management of residential centers, should consider this statement for the improvement of processes, internal communication, and employee motivation (10). At the moment, these quality systems take into account indicators such as family participation in activities, percentage of satisfied relatives or number of complaints received by the care home. But issues such as types of family members' involvement includes collaboration, family-staff relationship development, decision making and visiting are not explored in depth (41).

Advocacy of the residential center/home model and family involvement as indicators of quality are aligned to compliance with the 2030 SDGs. In particular, the data presented in this study points to SDG 16: transparency in institutions and ensuring responsive, inclusive and representative decision-making (42). This requires quality systems that create, enable and are geared to the fulfillment of these European/local commitments, particularly in the case of residential organizations or institutions whose CSR focus on the wellbeing of a vulnerable group. The collaboration between the business community and the academic sphere in the design of this multidisciplinary evaluation questionnaire has been of interest (43). Thus, the care home under study provided the object of analysis and the sample and the research group designed and validated the instrument. This work, therefore, is of interest to both parties: the care home has a measurement tool tailored to its services and to the demands of its customers and the research group has expanded its knowledge of social resources and vulnerable populations. CSR and knowledge transfer are synergies worth developing (44).

Despite its limitations—due to the small sample size and the survey methodology—the results of this study suggest practical implications for the design and implementation of strategies that benefit residents' quality of life and, by extension, their relatives' positive perception.

Future lines of research should focus on further innovation in the design of indicators of family satisfaction that can be collected during processes of internal quality evaluation, such as the UNE 158101 standard on the management of residential centers. A qualitative methodology, e.g., in-depth interviews or focus groups, would help to explore relatives' evaluation in greater detail. Considering the opinion of the care home professionals and neighbors in the community would also be of interest (45–47). Evaluation in the long term and in other settings (urban environment) would help to generalize the results to other times and contexts.

In any case, the objective of implementing these indicators is to encourage greater closeness between families and the staff at the center (management and medical service), and to discover their level of satisfaction regarding variables that determine user wellbeing. The improvement of the operational development of these indicators aims to ensure an integrated, multidimensional definition of the concept of wellbeing and quality of care.

## Data availability statement

The original contributions presented in the study are included in the article/supplementary material, further inquiries can be directed to the corresponding author.

## Ethics statement

Ethical approval was not required for the studies involving humans because all procedures performed in this study were in accordance with the ethical standards of the University of Zaragoza and with the 1964 Helsinki Declaration and its later amendments. The studies were conducted in accordance with the local legislation and institutional requirements. The participants provided their written informed consent to participate in this study. Written informed consent was obtained from the individual(s) for the publication of any potentially identifiable images or data included in this article.

## Author contributions

EL-M: Methodology, Validation, Visualization, Writing – original draft, Data curation. MS-G: Funding acquisition, Methodology, Validation, Visualization, Writing – review & editing. AG-L: Conceptualization, Data curation, Investigation, Methodology, Validation, Writing – original draft. MS-B: Conceptualization, Funding acquisition, Project administration, Resources, Visualization, Writing – review & editing. MG-L: Conceptualization, Methodology, Supervision, Validation, Visualization, Writing – original draft, Writing – review & editing.

## References

[1] Naciones Unidas. Valores Universales, Principio Dos: No Dejar a Nadie Atrás. (2019). Available online at: https://unsdg.un.org/es/2030-agenda/universal-values/leave-no-one-behind (accessed August 2, 2023).

[2] Balderrama-Tellez S Valerio-Vite N. La discapacidad y los Objetivos de Desarrollo Sostenible (ODS). Revista Legislativa. (2020) 27:75–102.

[3] Mc Donough W Braungart M. Desing for the triple top line: new tools for sustainable commerce. Corp Environm Strat. (2002) 9:251–8. 10.1016/S1066-7938(02)00069-6

[4] Comisión Europea. Comunicado de la comisión al Parlamento Europeo, al Consejo, al Comité Económico y Social Europeo y al Comité de las Regiones. Estrategia renovada de la UE para 2011-2014 sobre la responsabilidad social de las empresas. (2011). Available online at: https://eurlex.europa.eu/LexUriServ/LexUriServ.do?uri=COM:2011:0681:FIN:es:PDF (accessed August 2, 2023).

[5] Pfajfara G Shohamb A Małeckac A Zalaznikd M. Value of corporate social responsibility for multiple stakeholders and social impact – Relationship marketing perspective. J Bus Res. (2022) 143:46–61. 10.1016/j.jbusres.2022.01.051

[6] Fundación Edad & Vida. Guí*a de buenas prácticas para la integración de la RSE y los ODS de Naciones Unidas en el sector de servicios de atención a personas mayores*. (2017). Available online at: https://www.edad-vida.org/wp-content/uploads/2018/02/Gu'ıa-Buenas-Prácticas-integración-RSE-y-ODS-en-sector-Servicios-de-atención-a-personas-mayores.pdf (accessed August 2, 2023).

[7] Menichetti J Cipresso P Bussolin D Graffigna G. Engaging older people in healthy and active lifestyles: a systematic review. Ageing Soc. (2016) 36:2036–60. 10.1017/S0144686X15000781

[8] Dohmen MDW Huijg JM Woelders SMW Abma TA. Democratic care in nursing homes: responsive evaluation to mutually learn about good care. In:Spannring R Smidt W Unterrainer C, editors. Institutions and Organizations as Learning Environments for Participation and Democracy. Cham: Springer. (2022).

[9] Ley 5/2009 de 30 de junio de Servicios Sociales de Aragón. Boletín Oficial de Aragón ≪BOA≫ Núm. 132, de 10 de julio de 2009. Madrid: Agencia Estatal Boletín Oficial del Estado (2009).

[10] Asociación Española de Normalización y Certificación AENOR. Norma UNE 158101 de servicios para la promoción de la autonomía personal, gestión de centros residenciales y centros residenciales con centro de día o centro de noche integrado. In: Requisitos. Madrid: AENOR ediciones. (2007).

[11] Martínez Fernández R.; Barrera Algarín, E. Fortalezas y debilidades de los sistemas de gestión de la calidad implantados en los centros de personas mayores en España. Cultura de los Cuidados. (2021) 25:268–86. 10.14198/cuid.2021.61.17

[12] Saut AM Tobal F Moreno MC. Evaluating the impact of accreditation on Brazilian healthcare organizations: a quantitative study. Int J Qual Health Care. (2017) 29:mzx094. 10.1093/intqhc/mzx094 28992152

[13] García G Ramírez JM Aranda AM Rueda A. Ideas y propuestas para un nuevo modelo residencial para personas en situación de dependencia. In: Asociación Estatal de Directores y Gerentes en Servicios Sociales. (2021). Available online at: https://directoressociales.com/wp-content/uploads/2021/06/Modelo-residencias2021.pdf (accessed August 2, 2023).

[14] Koren MJ. Person-centered care for nursing home residents: the culture-change movement. Health Aff . (2010) 29:312–7. 10.1377/hlthaff.2009.0966 20056692

[15] Zimmerman S Shier V Saliba D. Transforming nursing home culture: evidence for practice and policy. Gerontologist. (2014) 54:5. 10.1093/geront/gnt161 24443601

[16] World Health Organization. Active Ageing: a Policy Framework. (2002). Available online at: https://apps.who.int/iris/handle/10665/67215 (accessed August 2, 2023).

[17] Finnema E De Lange J Droe ERM Ribbe M Van Tilbuerg W. The quality of nursing home care: Do the opinions of family members change after implementation of emotion-oriented care? J Adv Nurs. (2001) 35:728–40. 10.1046/j.1365-2648.2001.01905.x 11529975

[18] Hill NL Kolanowski AM Milone-Nuzzo P Yevchak A. Culture change models and resident health outcomes in long-term care. J Nurs Scholarsh. (2011) 43:30–40. 10.1111/j.1547-5069.2010.01379.x 21342422

[19] Shier V Khodyakov D Cohen LW Zimmerman S Saliba D. What does the evidence really say about culture change in nursing homes? Gerontologist. (2014) 54:gnt147. 10.1093/geront/gnt147 24443607

[20] Duan Y Mueller CA Yu F Talley KM. The effects of nursing home culture change on resident quality of life in US nursing homes: an integrative review. Res Gerontol Nurs. (2020) 13:210–24. 10.3928/19404921-20200115-02 31968121

[21] Shippee TP Henning-Smith C Gaugler JE Held R Kane RL. Family satisfaction with nursing home care: the role of facility characteristics and resident quality-of-life scores. Res Aging. (2017) 39:418–42. 10.1177/0164027515615182 26534835 PMC5777240

[22] Cooke H.; Puurveen, G.; Baumbusch, J. Exploring family involvement in resident care conferences in long-term residential care. Innov Aging, (2018) 2:396. 10.1093/geroni/igy023.1476

[23] Duan Y Mueller CA Yu F Talley KM Shippee TP. The relationships of nursing home culture change practices with resident quality of life and family satisfaction: toward a more nuanced understanding. Res Aging. (2021) 44:174–85. 10.1177/01640275211012652 33973498 PMC9126004

[24] Kellett UM. Transition in care: family carers' experience of nursing home placement. J Adv Nurs. (1999) 29:1474–81. 10.1046/j.1365-2648.1999.01035.x 10354243

[25] Zanetti O Geroldi C Frisoni GB Bianchetti A Trabucchi M. Contrasting results between caregiver's report and direct assessment of activities of daily living in patients affected by mild and very mild dementia: the contribution of the caregiver's personal characteristics. JAG. (1999) 47:196–202. 10.1111/j.1532-5415.1999.tb04578.x 9988291

[26] Poey JL Hermer L Cornelison L Kaup ML Drake P Stone RI. Does person-centered care improve residents' satisfaction with nursing home quality? J Am Med Dir Assoc. (2017) 18:974–9. 10.1016/j.jamda.2017.06.007 28754517

[27] Rodríguez-Martínez A Martín Cano M del C de la Fuente Robles YM Jiménez-Delgado JJ. Influence of facility characteristics on the quality of life of older adult residents: a systematic review. SAGE Open. (2023) 13:1–16. 10.1177/21582440231201001

[28] Campbell SM Roland M Buetow S. Defining quality of care. Soc Sci Med. (2000) 51:1611−25. 10.1016/S0277-9536(00)00057-5 11072882

[29] Zubritsky C Abbott KM Hirschman KB Bowles KH Foust JB Naylor MD. Health-related quality of life: Expanding a conceptual framework to include older adults who receive long-term service and supports. Gerontologist. (2023) 53:205–10. 10.1093/geront/gns093 22859435 PMC3695648

[30] Shippee TP Henning-Smith C Kane RL Lewis T. Resident and facility-level predictors of quality of life in long-term care. Gerontologist. (2015) 55:643–655. 10.1093/geront/gnt148 24352532 PMC4542585

[31] Leung M Yu J Chong MLA. Impact of facilities management on the quality of life for the elderly in care and attention homes: cross-validation by quantitative and qualitative studies. Indoor Built Environt. (2017) 26:1070–90. 10.1177/1420326X16662697

[32] Lucas JA Levin CA Lowe TJ Robertson B Akincigil A Sambamoorthi U Crystal S. The relationship between organizational factors and resident satisfaction with nursing home care and life. J Aging Soc Polic. (2007) 19:125–51. 10.1300/J031v19n02_07 17409050

[33] Miola L Carbone E Toffalini E Pazzaglia F. Navigability of residential care homes from residents', family members', and staff's points of view: the residential care home navigability scale. Gerontologist. (2023) 63:1419–27. 10.1093/geront/gnad029 36913365

[34] Degenholtz HB Kane RA Kane RL Bershadsky B Kling KC. Predicting nursing facility residents' quality of life using external indicators. Health Serv Res. (2006) 41:335–56. 10.1111/j.1475-6773.2005.00494.x 16584452 PMC1702527

[35] Cook KL Mayahara M Tivis L. Evaluation of the nurse practitioner offsite model. J Gerontol Nurs. (2023) 49:25–30. 10.3928/00989134-20230615-05 37379050

[36] Kehyayan V Hirdes JP Tyas SL Stolee P. Predictors of long-term care facility residents' self-reported quality of life with individual and facility characteristics in Canada. J Aging Health. (2016) 28:503–29. 10.1177/0898264315594138 26148943

[37] Arnoso A Pizarro M Arnoso M Asla N Elgorriaga E. Understanding loneliness and social exclusion in residential centers for social inclusion. Social Work Res. (2022) 46:242–54. 10.1093/swr/svac012

[38] Araya-Cuello M Fernández-Martínez M del M Hernández-Garre CM Carrión-Martínez JJ. Satisfacción de los adultos mayores con el programa socioeducativo de las Casas de Encuentro de Chile. CTS. (2020) 33:271–96. 10.5209/cuts.65232

[39] Avidor S Ayalon LI. Didn't meet my mother; i saw my mother“: the challenges facing long-term care residents and their families in the age of COVID-19. J Appl Gerontol. (2022) 41:22–9. 10.1177/07334648211037099 34365855

[40] Hayes BE. Cómo medir la satisfacción del cliente: diseño de encuestas, uso y métodos de análisis estadí*stico (No. 658834 H417co)*. México, MX: Alfaomega. (2009).

[41] Hayward JK Gould C Palluotto E Kitson E Fisher ER Spector A. Interventions promoting family involvement with care homes following placement of a relative with dementia: a systematic review. Dementia. (2022) 21:618–47. 10.1177/14713012211046595 34894796 PMC8811321

[42] Red Española del Pacto Mundial de Naciones Unidas. La misión del Pacto Mundial: 10 Principios + *17 ODS*. (2017). Available online at: https://www.pactomundial.org/noticia/10-principios-17-ods (accessed August 2, 2023).

[43] Ezzedine K Bennani M Shourick J Taieb C. A method for designing a patient burden questionnaire in dermatology. Clin Cosmet Investig Dermatology. (2020) 13:521–8. 10.2147/CCID.S260323 32821144 PMC7417926

[44] Mira-Aladrén M Martín-Peña Alonso-Hernando JB. Evaluación de la satisfacción en personas usuarias de actividades de envejecimiento saludable en Aragón. CTS. (2023) 36:317–32. 10.5209/cuts.84541

[45] Instituto Nacional de Seguridad y salud en el Trabajo INSST. In: *Red Española de Empresas Saludables*. (2021). Available online at: https://www.insst.es/red-espanola-de-empresas-saludables (accessed August 2, 2023).

[46] Gobierno de Aragón. Red Aragonesa de Empresas Saludables (RAES). (2021). Available online at: https://www.aragon.es/-/red-aragonesa-de-empresas-saludables (accessed August 2, 2023).

[47] Mira Tamayo DC Martínez Callaghan J Gil-Lacruz M. Valoración del a satisfacción de los servicios prestados en la residencia Alto Gállego. Zaragoza, ES: Grupo de Investigación Bienestar y Capital Social. (2019).

